# Increased body fat content in horses alters metabolic and physiological exercise response, decreases performance, and increases locomotion asymmetry

**DOI:** 10.14814/phy2.14824

**Published:** 2021-06-10

**Authors:** Anna Jansson, Vikingur Þ Gunnarsson, Sara Ringmark, Sveinn Ragnarsson, Denise Söderroos, Einar Ásgeirsson, Tanja R. Jóhannsdóttir, Charlotta Liedberg, Guðrún J. Stefánsdóttir

**Affiliations:** ^1^ Department of Anatomy, Physiology and Biochemistry Swedish University of Agricultural Sciences Uppsala Sweden; ^2^ Department of Equine Science Hólar University Sauðárkrókur Iceland

**Keywords:** body fat, hematocrit, lactate threshold, locomotion symmetry, performance

## Abstract

This study examined the effect of altered body weight (BW) and body fat content on exercise performance and recovery. Nine horses were divided into two groups, and changes in BW and fat content were induced by feeding a high (HA) or restricted (RA) energy allowance for 36 days in a cross‐over design. In the last week of each treatment, BW and body condition score (BCS) were recorded, body fat percentage was estimated using ultrasound, and a standardized incremental treadmill exercise test (SET) and competition‐like field test were performed (scored by judges blinded to treatments). Blood samples were collected, and heart rate (HR), rectal temperature (RT), and respiratory rate (RR) were also recorded. Objective locomotion analyses were performed before and after the field test. Body weight, body fat percentage, and BCS were higher (5–8%) in HA than in RA horses (*p* < 0.05). In SET, HA horses showed higher HR, plasma lactate concentration, RR, and RT than RA horses (*p* < 0.05), and lower V_La4_, hematocrit (Hct), plasma glucose, and plasma NEFA concentrations (*p* < 0.05). Hct was also lower in HA horses in the field test, while RA horses showed higher scores (*p* < 0.05). After both tests, resting plasma lactate concentrations were reached faster in RA than in HA horses (*p* < 0.05). Objective locomotion asymmetry was higher in HA than in RA (*p* < 0.05). These results clearly show that increased BW and body fat content in horses lower physiological fitness in terms of V_La4_, plasma lactate removal, Hct levels, plasma glucose availability and reduce true performance evaluated by blinded judges.

## Graphical abstract

The results clearly show that increased BW and body fat content in horses lower physiological fitness in terms of V_La_4, plasma lactate removal, Hct levels, plasma glucose availability and reduce true performance evaluated by blinded judges. Higher body fat content and BW alter metabolic and physiological responses to exercise by more than could be expected from increased weight load alone and it also cause locomotion asymmetry.

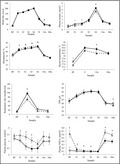

## 1 INTRODUCTION

In human athletes, it is generally accepted that body fat content can have marked effects on exercise performance, even in non‐overweight people (Lewis et al., 1986; Poortvliet et al., 2001). However, to our knowledge studies comparing exercise performance in the same individuals with different body fat conditions are missing. When comparing different groups of individuals, the results may easily be confounded by differences in genetics and training history. In horses, there is a lack of controlled studies using e.g., a cross‐over design, but field studies have examined the relationship between body condition, body composition, and performance in endurance horses (Garlinghouse & Burrill, 1999; Lawrence et al., 1992), Standardbred trotters (Kearns et al., 2002; Leleu & Cotrel, 2006), and Thoroughbred racehorses (Fonseca et al., 2013).

In a study on endurance horses, completion rates in 160‐km races were found to be highest in horses with body condition score (BCS) of 5–5.5 on a nine‐point scale, and no horses with BCS <3 completed the race (Garlinghouse & Burrill, 1999). In an earlier study on 2‐day endurance races (240 km), lower BCS was correlated with good performance (Lawrence et al., 1992). Other studies have shown a positive correlation between race time and body fat content in Standardbred horses (Kearns, McKeever, Kumagai, et al., 2002), a negative correlation between VO_2max_ and body fat percentage in Standardbred horses (Kearns et al., 2002), and a negative correlation between lactate threshold (V_La4_) and body fat percentage in French Trotters (Leleu & Cotrel, 2006). Higher fat‐free mass in Thoroughbred racehorses is suggested to be associated with good racing performance (Fonseca et al., 2013). A recent study on training responses by Klein et al., (2020) also showed lower body fat percentage and improved performance (run time to fatigue) in geldings compared to mares subjected to the same training program and ad libitum feeding. Whether these effects are due solely to altered weight‐bearing or partly to altered metabolism has not yet been clarified. However, it is known that body fat content affects circulating triglyceride and fatty acid levels, and insulin and glucose dynamics (Blaue et al., 2019; Hoffman et al., 2003; Jansson et al., 2016). An effect on exercise performance could therefore be expected.

The aim of the present study was to examine, using a cross‐over design, the effect of altered body fat content and body weight (BW) on exercise performance and recovery in horses. Higher body fat content and BW were hypothesized to impair performance, alter the metabolic and physiological response to exercise, and delay recovery. Increased weight bearing and fat deposits were hypothesized to have a negative effect on subjective gait performance and to increase locomotion asymmetry.

## 2 MATERIALS AND METHODS

The study was approved by the National Animal Research Committee of Iceland (no. 2016‐01‐01, case MAST1603119).

### 2.1 Experimental design

The study was conducted at Hólar University Collage in Iceland between 20 March and 2 June, 2016 and mean ambient temperature and humidity during the study was 4.2°C and 75%. Horses were divided into two groups (A and B, Table 1) and two diets were fed in a cross‐over design (cross‐over design), for 36 days each. It is well known that a 36 days adaptation period is enough to cause significant metabolic changes in horses (Connysson et al., 2010; Jansson & Lindberg, 2012; Ragnarsson & Jansson, 2011). The diets were called high energy allowance (HA; 64 ± 0.3 MJ metabolizable energy [ME]/day) and restricted energy allowance (RA; 32 ± 0.3 MJ ME/day). Both groups of horses had similar initial mean BW, body condition, age, girth circumference, and V_La4_ (*p* > 0.05) (Table 1). On specific days during the experiment (see below), all horses performed similar amounts of standardized exercise training. During the last week in each period, two exercise tests (see below) and locomotion asymmetry evaluations were performed.

**TABLE 1:** Mean body condition score^a^ (BCS), body weight (BW), girth circumference (GirthC), and lactate threshold (V_La4_) in the two groups of horses prior to entering the cross‐over study (mean ± SD)

	BCS	BW, kg	GirthC, cm	V_La4_, m/s
Group A	6.4 ± 0.2	401 ± 17	176 ± 4	5.4 ± 0.4
Group B	6.3 ± 0.2	401 ± 19	176 ± 5	5.2 ± 0.6

^a^ Back score excluded from the mean (see “Body condition and fat estimation”).

### 2.2 Horses

Nine geldings (6–8 years) of the Icelandic breed, with initial mean weight 401 ± 17 kg (mean ± SD) and BCS 6–6.6 (Henneke et al., 1983), were included in the study. All horses were at a similar stage in their training and were judged to be healthy and sound after a clinical examination by a veterinarian prior to the study. At the start of the study, the horses were clipped to partly remove the winter coat, to eliminate possible effects of variations in coat coverage (shift from winter to summer coat) on thermoregulation. They were housed separately from other horses, in individual 2.4 × 3 m (7.2 m^2^) stalls and kept outside in a paddock in two separate groups for 1–5 hr/day, depending on training and weather situations. In the week before the start of the study, all horses were familiarized with treadmill exercise and V_La4_ was determined (see below). During the study, the horses were trained five times/week by experienced riders. The training was performed in the same way and by the same rider, in all sessions and both periods for each horse. The horses wore heart rate (HR) recorders during all training sessions and mean training HR (122 ± 1 b.p.m. in HA; 120 ± 1 b.p.m. in RA) and training session duration (27 ± 0.4 min in HA; 26.5 ± 0.4 min in RA) did not differ between treatments (*p* > 0.05).

### 2.3 Feeding and management

The horses were fed three times/day (06.00, 15.00, and 21.30 hr) with a diet consisting of forage (10.7 ± 0.3 MJ ME/kg dry matter [DM], 163 ± 9 g/kg crude protein [CP]) and a commercial mineral and vitamin supplement (Racing Minerals, Trouw Nutrition). Mineral allowance was individually adjusted to the horse's requirements according to NRC (2007). The minerals were mixed with a commercial muesli (50 g/day, Besterly Herbic, Besterly Horse Feed) to increase palatability. Daily energy and protein intake in the two treatments are shown in Table 2. Water was available ad libitum, from water bowls (flow rate 7 L/min) in the boxes. Feed samples were gathered every other day and at opening of every new bale, pooled together weekly, and stored at −18°C until analysis. The haylage was analyzed for DM, ash, and CP as described by Palmgren‐Karlsson et al., (2000). The ME content was estimated in vitro (Lindgren, 1979).

**TABLE 2:** Daily dry matter (DM), metabolizable energy (ME), and crude protein (CP) intake in the high (HA) and restricted (RA) energy allowance treatments (LSM ± SE)

	HA	RA
DM, kg	5.9 ± 0.3	3.0 ± 0.1
ME/100 kg BW, MJ/day	17.0 ± 0.0	8.5 ± 0.2
CP, g/day	970 ± 45	483 ± 22

### 2.4 Body condition and fat estimation

Prior to the study and during the last week of each period (day 32), girth circumference was measured and the horses were weighed (Smartscale 300 Gallagher) and scored for body condition by a modified version of the system described by Henneke et al., (1983). The body was divided into sections (neck, back, rump, tail head, ribs, shoulder blade) and each section was given a score, using a continuous scale (Ringmark et al., 2013; Stéfansdóttir et al., 2017). Total BCS was then calculated as a mean of all sections. However, anecdotal evidence indicates that Icelandic horses do not necessarily accumulate fat along the back, and therefore two mean scores were calculated, one including back score and one without. In addition, during the last week of each period (day 32), neck circumference (tape measure), cresty neck score (Carter et al., 2009), and body fat percentage (fat%) were estimated using ultrasonic measurements (VET E Magic 2200, 5.0 MHz, with maximum depth set at 6.47, 5.0 MHz linear rectal probe, 80 elements, 4‐step multi‐frequency 3.5/5.0/6.0/7.0 MHz, Eickemeyer), as described by Westervelt et al., (1976). Subcutaneous fat thickness over the rump was measured 5 cm lateral to the midline at the center of the pelvic bone. The measurement site was shaved prior to every recording. Measurements were performed by the same person in both periods. The following equation was used (Ringmark et al., 2014; Westervelt et al., 1976) for estimation of body fat content:

Y=8.64+4.70x

where *Y* is body fat percentage and *x* is rump fat measurement in cm. Fat mass (FM) and fat free mass (FFM) were calculated by multiplying body weight with the estimated body fat percentage and by subtracting FM from body weight.

### 2.5 Exercise testing

During the week prior to the study, a standardized incremental exercise test (SET) was performed on a treadmill (Säto) to determine V_La4_ and individually adjust the velocity for the experimental SET. A portable blood lactate analyzer (Lactate Pro™ 2 LT‐1730m, Arkray Inc.) was used for the measurements. A SET was performed again during the last week of each treatment (day 29 or 30 in each period). SET results have been shown to be correlated with true performance in Warmblood riding horses (Bitschnau et al., 2010) and in trotters (Leleu et al., 2005). The test consisted of a 4‐min warm‐up in walk at 1.2 m/s with 0% incline, followed by four 2‐min incremental steps (S1–S4) with 6.25% incline and with mean speed per step of 3.5 ± 0.05, 4.3 ± 0.05, 5.0 ± 0.05, and 6.0 ± 0.05 m/s, respectively. Ambient temperature in the treadmill room was recorded after each SET (12.4 ± 0.4°C for HA, 11.6 ± 0.4°C for RA; *p* < 0.05).

The second exercise test was a field test simulating a breed evaluation field test (BEFT) for Icelandic horses, where riding abilities and gaits are scored by judges on a half‐point scale from 5 to 10. The test was performed according to regulations (FEIF, 2015) on treatment day 34, with the same experienced BEFT rider for all horses. The BEFT procedure was performed as described previously by Stefánsdóttir et al., (2014). The duration of the test was 10.7 ± 0.2 min and mean velocity was 4.18 ± 0.04 m/s (Polar G3, GPS sensor, Polar Electro). There were no differences in duration and velocity between treatments (*p* > 0.05). Mean ambient temperature during BEFT was 6.8 ± 0.1°C and mean wind speed was 3.2 ± 0.2 m/s (weather station: Model WH‐1080; Clas Ohlson). No differences were found in ambient temperature, wind speed, test duration, or mean velocity between treatments (*p* > 0.05). Horses were subjected to the same warm‐up (and same rider) in both treatments (duration: 9.4 ± 0.4 min for HA, 8.9 ± 0.4 min for RA; *p* > 0.05). In the first BEFT (first period), the rider chose the order and speed in which the gaits were ridden, according to what suited each horse. That structure was then repeated in the second BEFT (second period, second treatment). Two certified BEFT judges evaluated the horse in both BEFTs and were blinded to the treatment of the horse. All horses were shod according to regulations (FEIF, 2015).

Prior to both exercise tests, all horses were fed 1 kg DM of forage with the morning feed (06.00 hr), to minimize any potential effects of differences in gastrointestinal fill during the exercise tests. All exercise tests were performed between 09.00 and 16.00 hr, and for each individual always at the same time of the day.

### 2.6 Locomotion analysis

Locomotion asymmetry of each horse was assessed using the wireless sensor system Lameness locator (EQUINOSIS®, Columbia and St. Louis, MO; www.equinosis.com). The horses were fitted with the sensors according to McCracken et al., (2012). Recordings were conducted three times in each period; one day before BEFT and 1 and 2 days after BEFT. Recordings were performed on all horses while they were trotting in a straight line, both outside on a packed gravel road and inside on loose sand in a 20 × 60 m arena. The horses were trotted by hand for at least 50 m in each direction. Multiple recordings were performed if the trot was not clear, and recordings were considered for analysis if a minimum of 25 strides were included. Horses that had trouble trotting easily (shifting between trot, pace, and tölt) were fitted with 240 g heel‐weight riding boots to assist them in maintaining the gait. Whether boots were used or not was consistent on an individual basis for all recordings and in both treatments. Vector sum (VS) for both front limbs (VSF) and hind limbs (VSH) was calculated using the equation (Ringmark et al., 2016):

$$\text{VS}=\sqrt{{\left(\text{maximum difference}\right)}^{2}+{\left(\text{minimum difference}\right)}^{2}}$$

based on maximum and minimum height difference of the head and the pelvic sensors.

### 2.7 Data collection

During all training sessions, SET, and BEFT, HR (Polar HR Monitor RS800CX, Polar Pro Trainer 5 Equine Edition, equine H1 HR sensor, equine T56H transmitter W.I.N.D.), velocity, and distance (Polar G3, GPS sensor, Polar Electro) were recorded. In the morning of the experimental days, HR at rest was recorded using a stethoscope. During the SET, HR was recorded in the last 15 s of each step, at the end of a 2‐min cool‐down in walk, and 15 and 30 min post‐exercise. In BEFT, HR was recorded continuously during the test and 2, 15, and 30 min post‐exercise.

Respiratory rate (RR) was measured by counting the breaths for 15 s at rest, immediately after exercise, and 15 and 30 min post‐exercise. Rectal temperature (RT) was recorded at rest, after exercise, and 15 and 30 min post‐exercise (digital thermometer, digital thermometer, Omron, Healthcare Europe) in both SET and BEFT. RR and RT were recorded immediately after SET, but 2 min post‐exercise after BEFT.

### 2.8 Blood sampling and analysis

In SET, blood samples were collected from *vena jugularis*, using a jugular vein catheter inserted under local anesthesia (5 mL xylocaine 20 mg/mL; Astra Zeneca). Blood samples were collected before the test, in the last 15 s of every incremental step, and 15 and 30 min post‐exercise. Resting samples were taken at the same time for all horses in the box, 2 hr after morning feeding. In BEFT, blood samples were collected by jugular venipuncture before the test, 2 min after exiting the track, and 15 and 30 min post‐exercise. These samples were collected in chilled lithium heparin tubes (9 mL, Vacuette°; Greine‐Bio‐One) and stored on ice. From each blood sample, blood was collected into non‐heparinized capillary glass tubes and centrifuged for eight minutes (Cellocrit 2, AB Lars Ljungberg and Co) for hematocrit (Hct) analysis. Each sample was run in triplicate and the mean value was used for statistical analysis. Blood plasma was separated from all samples by centrifugation in heparin tubes for 15 min (520 *g*, EPA 12, Hettich zentrifugen) and stored at −18°C. An additional blood sample was taken at rest 2 days after BEFT, for analysis of aspartate amino transferase (AST) as a possible indicator of muscle leakage and/or damage and unaccustomed exercise (Mack et al., 2014). The AST was analyzed by an enzymatic method on a fully automated, open‐system clinical chemistry/immunoassay analyzer (Spectrophometer, Architect c 4000, Abbott Park). Total plasma protein (TPP) was analyzed using a refractometer (Atago). Blood plasma lactate concentration was analyzed in duplicate by an enzymatic method (Boehringer & Mannheims, lactat/r‐biopharmakit, kit no. 10139084035, Skandinavien Diagnostiska, intra‐assay CV <5%). Enzymatic methods were used to analyze (in duplicate) plasma glucose (D‐Glucose, kit no: E0716251, R‐Biopharm, Skandinavien Diagnostiska, intra‐assay CV <5%) and plasma non‐esterified fatty acids (NEFA) (Waco NEFA‐HR (1), (2), kits no. 434–91795 (RI) and 436‐91995 (R2), Nordic Biolabs, intra‐assay CV <4%) from blood samples collected before and during SET.

### 2.9 Statistical analysis and calculations

Calculation of V_La4_ was performed using Excel and an exponential curve fit. Statistical analysis was performed using R software (version 1.2.1335, The R Foundation for Statistical Computing). Normal distribution of data was confirmed with residual plots. Wilcoxon rank‐sum test was performed to analyze differences in initial BCS, BW, girth circumference, V_La4_, and age between the different groups of horses. The same test was used to analyze differences in daily feed intake between treatments.

The difference in training HR and duration between treatments was analyzed using a mixed model, *Y* = µ + *a_i_* + *b_j_* + *c_k_* + *d_l_* + *e_ijkl_*_,_ where *Y* is the observation, µ the mean value, *a_i_* the fixed effect of treatment, *b_j_* the fixed effect of period, *c_k_* the fixed effect of training session, *d_l_* the random effect of horse, and *e_ijkl_* the residuals. The effect of treatment on BW and BCS was analyzed using a mixed model, *Y* = µ + *a_i_* + *b_j_* + *d_l_* + *e_ijl_*, where *Y* is the observation, µ the mean value, *a_i_* the fixed effect of treatment, *b_j_* the fixed effect of period, *d_l_* the random effect of horse, and *e_ijl_* the residuals. The same model was used when analyzing ambient temperature in BEFT and SET, wind speed, duration, and velocity in BEFT, judges’ scores in BEFT, physiological parameters measured in BEFT and SET, and locomotion asymmetry data. When using this model, each sample was analyzed separately. When analyzing several samples together, sample was included in the model as a fixed effect. When comparing data between samples in each treatment, treatment and period were excluded from the model. When analyzing several days together, day was included in the model as a fixed effect. Surface (road or arena) did not affect locomotion asymmetry (*p* > 0.05) and was not included in the model. When comparing locomotion asymmetry between days in each treatment, treatment and period were excluded from the model.

Results are expressed as least square means (LSM) ± standard error (SE), and as mean ± standard deviation (SD), when stated. Tukey test was used for comparison, with the significance level set to *p* < 0.05 and the tendency level to *p* < 0.1. Correlations were determined using Pearson's correlation test.

## 3 RESULTS

All nine horses completed the study without clinical signs of injury, fatigue, or metabolic syndromes. Two horses had some days lost to training; one horse lost 3 days of training due to a lost shoe and another horse lost one day of training due to pastern dermatitis.

### 3.1 Body condition and body weight

Horses adapted to HA were heavier than horses adapted to RA (Table 3). Rump fat thickness, body fat percentage, FM, FFM and all parameters in body condition scoring except the score for back, cresty neck, and neck circumference were higher for HA than RA horses (Table 3).

**TABLE 3:** Body weight, rump fat thickness, body fat percentage (Fat %)^a^, fat mass (FM) and fat free mass (FFM)^b^, partial and mean body condition scores (BCS)^c^, and girth and neck circumference in nine horses after four weeks on a high energy allowance (HA) and a restricted energy allowance (RA) (cross‐over design, LSM ±SE)

Variable	HA	RA	*p*‐value
Body weight, kg	407 ± 5	388 ± 5	<0.0001
Rump fat thickness, mm	12.8 ± 0.3	11.5 ± 0.3	<0.005
Fat %	14.7 ± 0.1	14.0 ± 0.1	<0.005
FM, kg	60 ± 1	55 ± 1	<0.0001
FFM, kg	348 ± 4	334 ± 4	<0.0001
Neck score	6.1 ± 0.1	5.7 ± 0.1	<0.005
Back score	4.7 ± 0.1	4.6 ± 0.1	0.0622
Rump score	5.2 ± 0.1	5.1 ± 0.1	<0.05
Tail head score	5.9 ± 0.1	5.4 ± 0.1	<0.0001
Ribs score	6.9 ± 0.2	6.4 ± 0.2	<0.0001
Shoulder blade score	7.6 ± 0.2	7.0 ± 0.2	<0.0001
BCS_mean_	6.1 ± 0.1	5.7 ± 0.1	<0.0001
BCS_mean_ excl. back^d^	6.6 ± 0.1	6.1 ± 0.1	<0.0001
Cresty neck score^e^	2.3 ± 0.0	2.3 ± 0.0	0.8065
Girth circ., cm	175.0 ± 1.3	172.0 ± 1.3	<0.005
Neck circ., cm	104.0 ± 0.8	102.0 ± 0.8	0.1254

^a^ Estimated from ultrasound of the rump (Westervelt et al., 1976).

^b^ FM calculated by multiplying body weight with body fat percentage and FFM is body weight minus FM.

^c^ Henneke et al., (1983).

^d^ Back score excluded from the mean.

^e^ Carter et al., (2009).

### 3.2 Physiological and metabolic responses

#### 3.2.1 SET and VLa_4_

**FIGURE 1:**
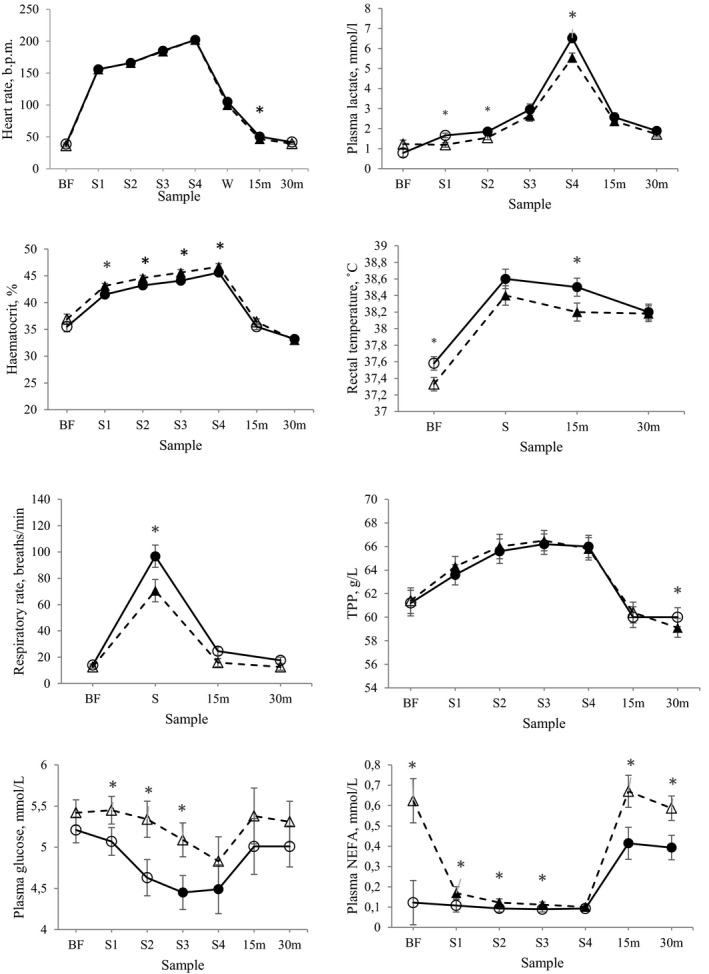
Physiological responses to a standardized, incremental exercise test (SET) in terms of heart rate (HR), plasma lactate concentration, hematocrit, rectal temperature, respiratory rate, total plasma protein concentration (TPP), plasma glucose, and plasma NEFA concentration, in nine horses subjected to high (HA, circles) and restricted (RA, triangles) energy allowance for one month in a cross‐over design (LSM ± SE). Samples were taken before (BF), at the end of each incremental step in SET (S1–S4), at the end of a 2‐min cool‐down in walk (W, only HR), immediately after the treadmill was stopped (S), and 15 and 30 min post‐exercise (15 m and 30 m). Filled markers indicate values that are significantly different from the previous value, Filled markers are significantly different from BF within treatment, * indicates significant difference between treatments (*p* < 0.05)

Heart rate increased linearly with speed during SET and was higher for HA compared with RA horses at 15 min post‐exercise (Figure 1). Horses in both treatments reached resting values 30 min post‐exercise (Figure 1). Plasma lactate concentration was higher in HA compared with RA throughout SET, and resting values were reached 30 min post‐exercise in RA, but not in HA (Figure 1). Lactate threshold was lower in HA compared with RA (5.4 ± 0.2 vs. 5.7 ± 0.2 m/s; *p* < 0.05). Hematocrit levels increased during SET and were lower for HA compared with RA (Figure 1). Resting values were reached 15 min post‐exercise in both treatments and values 30 min post‐exercise were lower than the resting values (Figure 1).

Rectal temperature was higher before SET and 15 min post‐exercise in HA compared with RA, and horses did not reach resting values by 30 min post‐exercise in either of the treatments (Figure 1). Respiratory rate immediately after exercise was higher for HA compared with RA, and horses reached resting values by 15 min post‐exercise in both treatments (Figure 1). Total plasma protein concentration 30 min post‐exercise was higher for HA compared with RA, and horses reached resting values by 15 min post‐exercise in both treatments (Figure 1). The TPP concentration in RA at 30 min post‐exercise was lower than the resting value (Figure 1).

Plasma glucose concentration in SET was higher in RA compared with HA in all SET steps except the last, and a decrease in plasma glucose concentration during SET was only observed in HA (Figure 1). The plasma NEFA concentration was higher in RA compared with HA horses for all observations except after the last step in SET (Figure 1). In RA, plasma NEFA decreased with exercise, but this was not observed in HA. However, there was a post‐exercise increase in plasma NEFA in both treatments (Figure 1).

#### 3.2.2 BEFT

**FIGURE 2:**
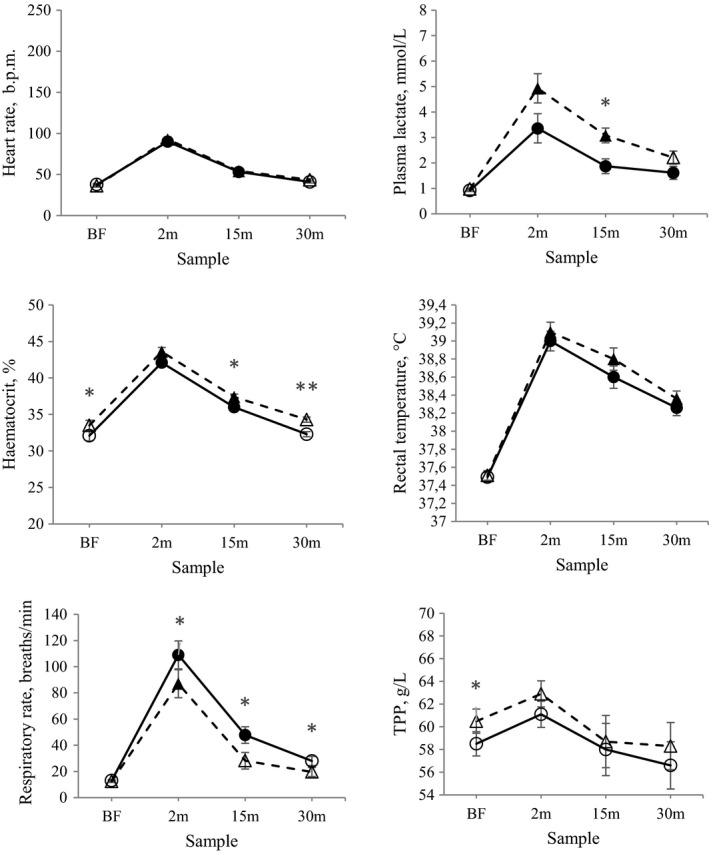
Physiological response to an exercise test (simulating a breed evaluation field test (BEFT) for Icelandic horses) in terms of heart rate, plasma lactate concentration, hematocrit, rectal temperature, respiratory rate, and total plasma protein concentration (TPP), in nine horses subjected to high (HA, circles) and restricted (RA, triangles) energy allowance for one month in a cross‐over design (LSM ± SE). Samples were taken before exercise (BF), 2 min post‐exercise, 15 min post‐exercise, and 30 min post‐exercise. Filled markers are significantly different from BF within treatment, * indicates significant difference between treatments (*p* < 0.05), and ** indicates significant difference between treatments (*p* < 0.01)

There was no effect of treatment on mean HR during BEFT (167 ± 2 b.p.m. for HA, 170 ± 2 b.p.m. for RA), before the test or at 2, 15, and 30 min post‐exercise (Figure 2). Horses in both treatments reached resting values by 30 min post‐exercise (Figure 2). Plasma lactate concentration was higher for RA compared with HA 15 min post‐exercise, but resting values were reached at 30 min post‐exercise in RA, but not in HA (Figure 2). The Hct was higher in RA compared with HA on all observation occasions except 2 min post‐exercise (Figure 2). Horses in both treatments had reached resting values 30 min post‐exercise.

Rectal temperature in BEFT did not differ between treatments and resting values were not reached 30 min post‐exercise (Figure 2). Respiratory rate was higher in HA compared with RA post‐exercise, and in RA resting values were reached 15 min earlier than in HA (Figure 2). Total plasma protein was higher in RA than in HA horses before exercise, but did not differ between treatments post‐exercise or from the resting value (Figure 2).

### 3.3 Locomotion analysis and AST response

**FIGURE 3:**
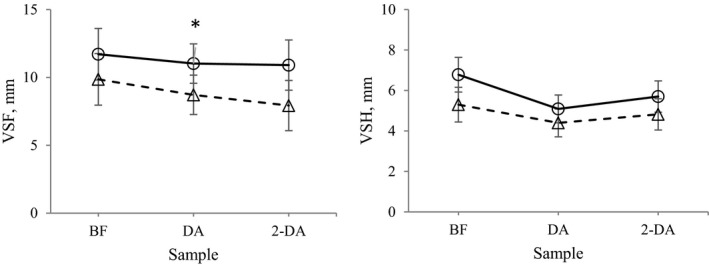
Locomotion asymmetry (vector sum front (VSF) and hind (VSH)) in horses subjected to high (HA, cicles) and restricted (RA, triangles) energy allowance for one month in a cross‐over design (LSM ± SE). Measurements were performed one day before (BF), during exercise simulating a breed evaluation field test (BEFT), and one and two days after BEFT (DA and 2‐DA, respectively). Filled markers are significantly different from BF, * indicates significant difference between treatments (*p* < 0.05)

Overall front limb asymmetry, expressed as VSF, was higher in HA horses compared with RA horses (11.2 ± 1.6 mm vs. 8.8 ± 1.6 mm; *p* < 0.05), and for individual days the difference was significant one day after BEFT (Figure 3). Overall VSH was higher in HA than in RA (5.9 ± 0.6 mm vs. 4.8 ± 0.6 mm; *p* < 0.05), but was not different on individual days (Figure 3).

There was no difference in AST activity before BEFT and two days after BEFT (*p* > 0.05). There was also no difference between treatments (before: 5.5 ± 0.6 ukat/L in HA vs. 5.3 ± 0.6 ukat/L in RA, two days after: 5.2 ± 0.6 ukat/L in HA vs. 5.1 ± 0.6 ukat/L in RA) (*p* > 0.05).

### 3.4 Performance in BEFT

Judges’ scores for gallop, form under rider, and total score for riding abilities were lower (*p* < 0.05) for HA than for RA (Table 4). Scores for tölt and pace showed a tendency to be lower for HA than for RA (*p* = 0.09 and *p* = 0.06, respectively) (Table 4).

**TABLE 4:** BEFT^a^ scores (LSM ± SE) awarded by blinded judges to nine horses subjected to a high energy allowance (HA) and restricted energy allowance (RA) for 4 weeks in a cross‐over design

	HA	RA	SE	*p*‐value
Tölt	7.09	7.41	0.18	0.0861
Slow tölt	6.92	7.14	0.24	0.3868
Trot	7.11	7.28	0.31	0.2547
Pace	5.10	5.29	0.13	0.0642
Gallop	7.62	7.83	0.14	<0.05
Canter	7.44	7.44	0.11	1.0000
Spirit	7.43	7.62	0.14	0.2773
Form under rider	7.44	7.67	0.13	<0.05
Walk	7.65	7.57	0.20	0.5882
Total riding abilities^b^	6.95	7.17	0.09	<0.05

^a^ An exercise test simulating a breed evaluation field test for Icelandic horses.

^b^ Calculated from the above scores (FEIF, 2015).

## 4 DISCUSSION

To our knowledge, this is the first study to examine the effect of increased body fat content and BW on metabolic and physiological exercise response and performance in horses in a controlled cross‐over study. The results clearly show that an increase in body fat percentage, BW, and body condition of 5–8% lowers physiological and metabolic fitness in terms of V_La4_, plasma lactate removal, Hct levels, plasma glucose availability, and recovery of RR. It also impairs true performance evaluated by judges blinded to the treatments, and appears to induce locomotion asymmetry, measured objectively using a sensor technique.

As expected, horses in the HA group had higher HR, plasma lactate, RR, RT, and TPP concentrations than RA horses in one or several samples during SET, indicating higher workload and exercise intensity. However, further analysis showed that the metabolic and physiological alterations and reduction in performance were most likely due not only to increased workload, but also to adaptive physiological and metabolic changes, or even dysfunctions. The elevated plasma NEFA concentration prior to exercise and the marked drop during exercise in RA horses, together with the lack of drop in glucose concentrations, show that the horses were efficiently adapted to fat utilization on RA and less dependent on glucose during exercise compared with HA horses. This implies that aerobic pathways (citric cycle) must have been promoted in RA horses, as also suggested by the improvement in V_La4_. Lowered plasma glucose concentration, as in HA horses, may also have had negative impacts on performance, since it has been shown that plasma glucose infusion during submaximal exercise can increase the duration of exercise (Farris et al., 1998).

Although BEFT was performed outdoors in field conditions, the majority of the physiological responses were the same as in SET. In one exception, the response in plasma lactate concentration was different between the exercise tests, with horses on RA responding with higher levels than HA horses in BEFT, not lower as in SET. This is not surprising, since the horses performed better on RA (higher scores) and higher scores have been associated with higher plasma lactate levels in BEFT (Stefánsdóttir et al., 2014). Speed is an important determinant of the scores in several of the gaits evaluated in BEFT, with the higher the speed, the better the score (Stefánsdóttir et al., 2021). Higher lactate in the RA horses might be explained by higher speed within one or more gaits, although the mean speed during the whole BEFT did not differ between treatments.

Rectal temperature response did not differ between treatments during BEFT, but it did during SET. The reason for this is unclear. Following SET, RT was higher in HA and the elevation was most likely a response to the increased workload and heat production, but it is unclear why a difference was observed already before exercise. The increase in RR post‐exercise in both tests can also have been caused by the increased workload and differences in body temperature. Interestingly, TPP was higher in HA than in RA 30 min post‐SET, indicating that there may have been a delay in the recovery of exercise‐induced fluid losses in HA horses.

The recovery pattern of plasma lactate levels was similar in both SET and BEFT, i.e. it was slower in HA than in RA horses. This indicates that either lactate removal from the blood and/or storage in red blood cells was limited, or that the enzymatic capacity to metabolize lactate was lower. Interestingly, Hct was lower in HA than in RA horses in both SET and BEFT. One could hypothesize that exercise Hct was higher in HA due to the higher workload (higher BW, Nyman et al., 2002), but we observed the opposite. It has been shown previously that increased forage allowance, as in the present study, may cause a decrease in TPP (indicating dilution of the plasma volume) (Connysson et al., 2010; Danielsen et al., 1995; Jansson & Lindberg, 2012). A possible consequence of this could be lowering of Hct. Total plasma protein concentration was lower in HA during BEFT, but not during SET, so it seems not to be the sole explanation for the reduced Hct in the present study.

The impact of hypercaloric diets and obesity on spleen function and red blood cells is largely unexplored (Unruh et al., 2015). However, the red pulp of the spleen is a site of erythrophagocytosis and a hypercaloric diet modulates splenic function and red blood cell externalization in mice, promoting interaction with erythrophagocytosis macrophages (Unruh et al., 2015). Those authors concluded that red blood cell dysfunction occurs early (within weeks) during diet‐induced obesity. In a later study comparing mice on a hypercaloric diet with controls, spleen size was found to be increased and more macrophages were observed in the spleen of mice on the hypercaloric diet (Buchan et al., 2018). We therefore suggest that the gain in body fat in the present study increased red blood cell phagocytosis, which contributed to the lower Hct. This may be the first observation of altered red cell function in horses due to diet‐induced weight gain.

### 4.1 Locomotion and true performance

Overall, locomotion asymmetry was higher in HA than in RA horses and was most pronounced in front limbs one day after BEFT. This indicates that weight gain may have a negative impact on the locomotion apparatus and that the effect may be amplified after acute exercise. Previous studies have shown a relationship between performance and asymmetry. For instance, lower locomotion asymmetry in 4‐ or 5‐year‐old Warmblood horses increases their overall lifetime performance and number of years active in competitions (Jönsson et al., 2014). Low front limb asymmetry in trotters has been associated with early qualification for races, indicating greater athletic abilities (Ringmark et al., 2014). The reason for the altered locomotion pattern in the present study is unclear. However, it is known from studies in humans that obesity changes biomechanics at walk (Browning, 2012) and that weight loss may decrease musculoskeletal pain (McGoey et al., 1990). In dogs with osteoarthritis, a weight loss of 6% or more can achieve a significant reduction in lameness (Marshall et al., 2010). Besides the extra load caused by obesity, metabolic‐triggered inflammation, where adipose tissue produces inflammatory mediators, is known as a major contributing factor to osteoarthritis in humans (Berenbaum et al., 2013). However, no such relationship has yet been confirmed in horses, although inflammatory cytokine production has been found to increase with increasing body fat content in aged horses (Adams et al., 2009).

The levels of AST recorded indicate that muscle tissue leakage or damage was not a problem, since BEFT per se, HA, and an increase in asymmetry were not linked to elevated AST concentrations. Similar results have been reported in a study evaluating the effects of increased weight carrying in horses (up to 35% of BW, using lead weights) (Stefánsdóttir et al., 2017).

We observed a decrease in VLa_4_ as well as in true performance with the HA treatment but in a recent study it has been suggested that horses can maintain runtime to fatigue performance with a moderate weight gain (~5% body fat percentage) if they are given a long enough training period (Klein et al., 2020). Further studies are needed to evaluate the effect on performance of long term (months) differences in body condition but studies on competing horses nevertheless indicate that there is a discipline specific range where body condition is optimal (Garlinghouse & Burrill, 1999; Lawrence et al., 1992; Leleu & Cotrel, 2006).

To achieve the higher body fat content horses on HA were overfed. The increase in BW was therefore not only due to increased FM but to some extent also to an increased content of the gastrointestinal tract (indigestible material and water) as well as an increase in the plasma volume (Connysson et al., 2010; Danielsen et al., 1995; Jansson & Lindberg, 2012). The body fat percentage calculation indicate that FM was increased by some 5 kg and that the remaining change in BW (14 kg) was something else. The accuracy of the fat percentage calculation for Icelandic horses is unknown, since the equation is developed from analyses of horses of unknown breed, but based on other studies were the forage allowance has been increased substantially, 14 kg is likely a substantial overestimation of gastrointestinal fill. In several studies were a high concentrate allowance (4–6 kg) has been exchanged with forage with the same high energy content as in the present study, i.e. the forage allowance has been doubled, a weight increase of 3 kg has been observed in 500 kg horses (Connysson et al., 2010; Jansson & Lindberg, 2012). Based on daily ME intake and expected ME requirements we estimate that horses were fed a surplus of 13–18 MJ ME every day on HA (fed 68 MJ ME but required approximately 50–55 MJ ME, Jansson et al., 2011), which during a period of 30 days corresponds to approximately 10–15 kg of fat (1 kg of fat=39 MJ ME). The accuracy of the fat percentage calculation can therefore be questioned by several reasons. It is likely that there is a difference between individuals and possibly breeds in fat accumulation patterns, i.e. in the proportion of fat accumulated subcutaneously, intramuscular and visceral. Nevertheless, the method has earlier been shown useful in for example Standardbred horses (Klein et al., 2020) and in the same breed rump fat thickness has also been negatively correlated with VLa_4_ (Ringmark et al., 2017).

### 4.2 Relevance to the Icelandic horse community

Although the results of the present study are of relevance for all types of horses and perhaps most mammals, the BEFT response is of special relevance for horses of the Icelandic breed and its users. There are approximately 280,000 individuals of this breed, and it is found in >30 different countries world‐wide (WorldFengur, 2020). The Icelandic horse is considered to be an “easy keeper” and is prone to maintaining positive energy balance (Ragnarsson & Jansson, 2011). Jensen et al., (2016) found that 24% of Icelandic horses in Denmark were overweight or obese. Increased knowledge about the effects of increased body fat content is therefore of extreme importance for this breed. Interestingly, the change in locomotion pattern detected with an objective method seemed also to be visible to the eye, as the judges’ scores for some gaits were lower for horses on HA. There was also a significant effect on “form under rider,” where horses on HA made a less positive impression. We have previously demonstrated a positive correlation between high scores in true BEFT and physiological parameters linked to high aerobic capacity (Stefánsdóttir et al., 2014). Altogether, this provides good reason for trainers of Icelandic horses to pay attention to body condition in their horses when striving for maximum performance. The results from our body condition scoring also confirm anecdotal observations that Icelandic horses do not primarily accumulate fat over the back, at least not in this range (5.7–6.1) of the scoring system. Scoring of ribs and tail head may therefore be more accurate than scoring the back to monitor weight gain or loss at these body condition levels. The use of Henneke et al., and and’s (1983) scoring system on Icelandic horses can be questioned since this system was originally developed on another breed. However, we have used this system earlier and observed a correlation between scores and plasma insulin levels in a group of both Icelandic and Standardbred horses (Ragnarsson & Jansson, 2011) as well as a correlation to the lactate threshold in Icelandic horses (Stefánsdóttir et al., 2017), indicating that the system reflects alterations in the metabolism irrespective of breed.

### 4.3 Period effects

Elevated HR during exercise was expected in HA, but was not observed until 15 min post exercise. This may have been due to a higher level of excitement during the first test, which is supported by the effect of period on HR, and on Hct and glucose, observed during SET, with higher mean values in the first period. The horses had been trained for the test, but they might have needed more preparation. The suggestion that test and the preparations prior to the test were associated with some excitement is also supported by the slightly elevated resting Hct prior to exercise (~36%) compared with 30 min post‐exercise (~32%). The horses were familiarized with the treadmill in 3–4 sessions prior to the study, which is more than the 1–2 sessions recommended by King et al., (1995). To minimize the influence of stress on the physiological response to treadmill exercise, horses may need to be trained on the treadmill more than 3–4 times.

## 5 CONCLUSIONS

Higher body fat content and BW in horses was shown to alter metabolic and physiological responses to exercise by more than could be expected from increased weight load alone. Higher body fat content and BW in horses also lowered true performance, caused locomotion asymmetry, and delayed recovery from exercise.

## AUTHOR CONTRIBUTION

Jansson, Stefánsdóttir, Gunnarsson and Ragnarsson designed the frames of the work. Jansson, Stefánsdóttir, Gunnarsson, Ringmark, Ásgeirsson, Jóhannsdóttir and Liedberg were responsible for details in the design and for the acquisition of data. All authors were responsible for analysis, and/or interpretation of the data. Jansson and Söderroos were responsible for drafting the work or revising it critically for important intellectual content.
